# Comparison of the associations between non-traditional and traditional indices of adiposity and cardiovascular mortality: an observational study of one million person-years of follow-up

**DOI:** 10.1038/s41366-019-0353-9

**Published:** 2019-03-29

**Authors:** Anne Pernille Ofstad, Christine Sommer, Kåre I Birkeland, Marit Rokne Bjørgaas, Jon Michael Gran, Hanne Løvdal Gulseth, Odd Erik Johansen

**Affiliations:** 1 Department of Medical Research, Bærum Hospital, Vestre Viken Hospital Trust, Gjettum, Norway; 2 Medical Department, Boehringer Ingelheim Norway, Asker, Norway; 3 Department of Endocrinology, Morbid Obesity and Preventive Medicine, Oslo University Hospital, Oslo, Norway; 4 Department of Transplantation Medicine, Oslo University Hospital, Rikshospitalet, Norway; 5 Faculty of Medicine, University of Oslo, Oslo, Norway; 6 Department of Clinical and Molecular Medicine, Faculty of Medicine and Health Sciences, NTNU, Norwegian University of Science and Technology, Trondheim, Norway; 7 Department of Endocrinology, St. Olav’s Hospital, Trondheim University Hospital, Trondheim, Norway; 8 Oslo Centre for Biostatistics and Epidemiology, Oslo University Hospital and University of Oslo, Oslo, Norway

**Keywords:** Cardiovascular diseases, Obesity, Risk factors

## Abstract

**Background/objective:**

The most widely used adiposity index, body mass index (BMI), is not optimal to evaluate cardiovascular (CV) risk associated with overweight and obesity. We aimed to explore the association between traditional and non-traditional adiposity indices and CV mortality, and compare their discriminative ability for CV death.

**Methods:**

We studied participants (age 19–79 years, BMI ≥18.5 kg/m^2^) from the population-based Norwegian Nord-Trøndelag Health Study 2 (HUNT 2). Traditional indices explored were BMI, waist circumference (WC) and waist- to-hip ratio, whereas non-traditional were estimated total body fat (eTBF), which is a sex-specific fat%-index, index of central obesity (WC/height) and a body shape index (ABSI) [WC/(BMI^2/3^ × √height)]. Associations between the traditional and non-traditional indices and CV death, obtained from the Norwegian Cause of Death Registry, were explored by Cox proportional hazard regression, and the indices’ discriminative ability by Harrell’s *C* statistics.

**Results:**

Baseline assessments were done from 1995 to 1997 and the population (*n* = 61,016, 52% women) was observed for 17.7 ± 4.2 years (until 2016), yielding 1,080,473.6 person-years of follow-up. Thirteen thousand one hundred and ninety five (21.6%) subjects died, of whom 4908 (37.2%) died from CV causes. Across genders, eTBF had the strongest association to CV death (unadjusted hazard ratios [HRs] 4th vs. 1st quartile in women and men 13.38 [95% confidence interval (CI): 11.05–16.22] and 9.32 [8.03–10.81], respectively), together with index of central obesity in women and ABSI in men. The other indices showed weaker associations, in particular BMI in men: 1.73 [1.56–1.93]. Age adjustment attenuated the associations, but the pattern remained. In concordance with this, *C*-statistics was *C* = 0.725 [0.713–0.737] in women and 0.711 [0.701–0.721] in men for eTBF, and *C* = 0.622 [0.610–0.634] in women and 0.551 [0.541–0.562] in men for BMI.

**Conclusion:**

eTBF, a sex-specific total body fat index, was more strongly associated with CV death than other adiposity indices and may be a suitable clinical tool for assessment of obesity-associated CV risk.

## Introduction

The prevalence and disease burden of overweight and obesity is increasing, and data from 1990 to 2015 suggest that obesity is related to nearly four million deaths globally, of which cardiovascular (CV) deaths account for nearly 70% [1]. Therefore, the various scientific societies recommend to avoid unhealthy fat accumulation for optimal health in most people [2].

Body mass index (BMI) is the most widely used standardised index to define normal weight, overweight and obesity in clinical practice [3]. BMI is easy to use, but it does not distinguish between lean body mass and fat mass and its distribution (visceral vs. subcutaneous), and it does not account for biological gender differences in fat function and distribution. Fat mass and its distribution, particularly abdominal fat, is strongly associated with adverse outcomes and data suggest that intra-abdominal (visceral) adipose tissue may be a primary driver of the cardiometabolic complications of obesity [4].

Most epidemiological studies that include measures of validated indices of body fat or body fat distribution show that these appear to predict CV complications more precisely than BMI [5–8]. Traditional indices of adiposity include waist circumference (WC) and waist-to-hip ratio (WHR), whereas non-traditional, and less studied, indices include WHR (often called index of central obesity, ICO) [7, 9], a body shape index (ABSI) reflecting abdominal visceral fat [10] and estimated total body fat (eTBF), which is based on the Young Man’s Christian Association’s (YMCA) gender-specific formulas [11, 12].

The aim of this study was to explore the associations of readily available non-traditional (eTBF, ABSI, ICO) and traditional (WC, WHR, BMI) anthropometric indices of fat and fat distribution with long-term CV mortality in the large population-based The Nord-Trøndelag Health Study 2 (HUNT 2) study, and to contrast their ability to predict CV mortality risk in order to identify the best adiposity index for routine clinical use.

## Materials and methods

### Study population

HUNT 2 was the second wave of the original HUNT study [13], a population-based multi-purpose health study in the ethnically homogenous county of Nord-Trøndelag, Norway. This population is considered representative of the total ethnic Norwegian population regarding demography, socio-economic factors, morbidity and mortality, including mortality from CV disease [14]. For HUNT 2, all residents 20 years or older were invited to a screening visit between August 1995 and July 1997. Subjects responded to a comprehensive health and lifestyle questionnaire and underwent a general health examination. Details of the protocol and study design have been described previously [14]. For the purpose of this study, we excluded individuals with BMI <18.5 kg/m^2^ and those with age <19 or >79 years.

### Anthropometric measures, indices of adiposity and laboratory assessments

All exposures were assessed using standardised methodology. Body height and body weight were recorded with the participants wearing light clothes and no shoes. Height was measured to the nearest centimetre and weight to the nearest 0.5 kg. Based on these measures, BMI was calculated as weight (in kg) divided by the squared value of height (in m). WC and hip circumferences were measured with a steel band to the nearest centimetre with the participant standing and with the arms hanging relaxed. The WC was measured horizontally at the height of the umbilicus, and the hip circumference was measured likewise at the thickest part of the hip [14].

ICO was calculated as WC/height [7, 9], WHR as WC/hip circumference, eTBF by the sex-specific YMCA formulas (men: 100 × (−98.42 + [4.15 × WC (in.)] – [0.082 × weight (lbs)])/weight; females: 100 × (−76.76 + [4.15 × WC] – [0.082 × weight])/weight) [11, 12] and ABSI as WC [m]/(BMI^2/3^ × √height [m]) [10].

Systolic and diastolic blood pressure was measured three times and the mean of the two last measurements was used. All blood samples were drawn in the non-fasting state. Participants who either reported to have diabetes mellitus or who had a non-fasting blood glucose above 11.1mL/L at baseline (i.e. at inclusion in HUNT 2) were defined as having diabetes. CV comorbidities at baseline were self-reported by participants (previous myocardial infarction, episode of angina pectoris, or stroke), as were smoking habits (“no smoking”, “former smoking” or “current daily smoking of cigarettes, cigars or pipe”). Education was categorised into having completed: primary school, 1–2 years of secondary school, high school, <4 years of college/university or >4 years of college/university.

### Follow-up and outcome

All participants enrolled in HUNT 2 (1995–1997) included in the present study were followed for CV mortality. Using the participants’ unique personal identity number, HUNT 2 data were linked to the Norwegian Cause of Death Registry, to which all deaths in Norway are reported. Death from CV disease was defined according to the International Classification of Disease codes ICD-9: 390–459 for deaths before 2005 and ICD-10: 100–199 for deaths as of 1 January 2005. Subjects were followed until death or until 31 December 2015, whichever occurred first.

### Informed consent and ethics

All participants in HUNT 2 gave written informed consent and the study was approved by the Regional Committee for Medical and Health Research Ethics and by the Norwegian Data Inspectorate. The present study and protocol were approved by the Regional Committee for Medical and Health Research Ethics in 2016.

### Statistical analyses

All adiposity indices were categorised into sex-specific quartiles. We used Cox proportional hazard models to explore the associations between the different categories of the indices of adiposity and CV death, with the lowest quartile as the reference category. The proportional hazard assumption was assessed by a test based on Schoenfeld’s residuals. We analysed data from men and women separately in crude models as well as in models adjusted for age. To address potential confounding factors, we conducted sensitivity analyses in which we excluded participants with known CV disease, diabetes or current smoking, respectively.

Harrell’s *C*-statistics were calculated based on the Cox proportional hazard regression models to assess individual indices’ discrimination [15]. C- index is developed for survival analysis and describes the adiposity index’ ability to distinguish between persons with longer event-free survival and those with shorter event-free survival within a given time horizon [16]. The C-index ranges from a minimum of 0.5 (no discriminatory accuracy) to a theoretical maximum of 1.0 (perfect discrimination). To explore the continuous relationship between the adiposity indices and CV death, we used fractional polynominal plots displaying the HR for CV death for each unit of the adiposity index, for men and women separately. Owing to very low number of participants in the highest ranges of the indices, the graphical displays exclude the 0.5% highest and lowest values for men and women for each adiposity index. We used sex-specific *z*-scores to compare HR for CV death across different adiposity indices.

We analysed relationships between CV mortality and BMI, WC, ICO, WHR, eTBF and ABSI by quartiles of adiposity indices, with the lowest quartile as the reference category. To reduce the influence of competing risk, we excluded those with age ≥80 years and BMI <18.5 kg/m^2^, and due to gender differences in fat mass distribution/function, we also investigated this relationship for women and men separately. Further, since age and frailty may influence the relationship, we also analysed the associations in two age strata: younger: 19–59 years vs. older: 60–79 years. Absolute risk of CV death was expressed as sex-specific incidence rates per 1000 patient year per quartile of the adiposity indices.

Analyses were performed using SPSS version 24.0 (SPSS Inc., Chicago, IL, USA), STATA version 14 (StataCorp, College Station, TX, USA), and R (R Core Team (2017). R: A language and environment for statistical computing. R Foundation for Statistical Computing, Vienna, Austria. URL https://www.R-project.org/). In all analyses, *p* < 0.05 was considered significant.

## Results and discussion

### Population characteristics

Of 92,434 eligible subjects in Nord-Trøndelag County, 66,140 men and women (71.2%) attended the screening. Excluding individuals with BMI <18.5 kg/m^2^ and not within age-span 19–79 years rendered 61,016 individuals (31,936 women, 29,080 men) to be included in this study, and baseline characteristics are given in Table 1. Male participants, as compared to female participants, had higher weight (83.5 ± 12.2 vs. 70.8 ± 12.5 kg), systolic and diastolic blood pressure (140 ± 19 vs. 135 ± 23 and 82 ± 12 vs. 79 ± 12 mmHg, respectively), *s*-triglycerides (1.9 ± 1.1 vs. 1.5 ± 0.9 mmol/L) and eGFR (96.9 ± 19.8 vs. 92.7 ± 20.9 mL/min/1.73^2^). A larger proportion of men had previous CV disease (8.9 vs. 5.0%). The mean BMI was similar in women and men, whereas the other adiposity indices differed between genders, with the most notable difference observed for the mean WC, which was higher in men (91.9 ± 9.3 vs. 81.5 ± 11.4 cm), and the mean eTBF which was higher in women (19.5 ± 5.5 vs. 27.3 ± 7.4%). In both sexes, there were significant correlations between all adiposity indices (Supplementary Table 1).

**Table 1:** Baseline characteristics of the study population by sex

	Women (*n*=31,936)		Men (*n*=29,080)	
		*N* with available data		*N* with available data
Age (years)	48.8 ± 16.1	31,936	48.7 ± 15.8	29,080
DM, *n* (%)	898 (2.8%)	31,936	951 (3.3%)	28,975
CV disease, *n* (%)^b^	1579 (5.0%)	31,849	2592 (8.9%)	29,057
Weight (kg)	70.8 ± 12.5	31,936	83.5 ± 12.2	29,080
Systolic BP (mmHg)	135 ± 23	31,861	140 ± 19	28,963
Diastolic BP (mmHg)	79 ± 12	31,851	82 ± 12	28,955
Cholesterol (mmol/L)^a^	5.9 ± 1.3	31,713	5.8 ± 1.1	28,976
Glucose (mmol/L)^a^	5.3 ± 0.9	31,560	5.4 ± 1.0	28,604
Triglycerides (mmol/L)^a^	1.5 ± 0.9	31,838	1.9 ± 1.1	28,905
eGFR (mL/min/1.73 m^2^) (MDRD)	92.7 ± 20.9	31,845	96.9 ± 19.8	28,998
Smoking status		31,225		28,626
Never smokers	14,673(47.0%)		10,840 (37.9%)	
Former smokers	7004 (22.4%)		9456 (33.0%)	
Current daily smokers	9548(30.6%)		8330 (29.1%)	
Unknown/missing	711 (2.2%)		454 (1.6%)	
Education		30,312		27,884
Primary school	11,857(39.1%)		8646 (31.0%)	
1–2 years of high school	8950 (29.5%)		11,307 (40.6%)	
Junior college	3284 (10.8%)		2283 (8.2%)	
College/university (<4 years)	4014 (13.2%)		3287 (11.8%)	
College/university (4 years or more)	2207 (7.3%)		2361 (8.5)	
Adiposity indices				
Waist circumference (cm)	81.5 ± 11.4	31,936	91.9 ± 9.3	29,080
Waist-to-hip ratio	0.80 ± 0.06	31,934	0.90 ± 0.06	29,079
BMI (kg/m^2^)	26.3 ± 4.5	31,936	26.5 ± 3.5	29,080
ICO	0.50 ± 0.07	31,936	0.52 ± 0.05	29,080
eTBF (%)	27.3 ± 7.4	31,936	19.5 ± 5.5	29,080
ABSI	0.72 ± 0.05	31,936	0.78 ± 0.04	29,080

*SD* standard deviation, *DM* diabetes mellitus, *eGFR* estimated glomerular filtration rate, *MDRD* modified diet in renal disease, *BMI* body mass index, *ICO* index of central obesity, *eTBF* estimated total body fat, *ABSI* a body shape index

Data are given as mean plus/minus SD or proportions

^a^Non-fasting

^b^Self-reported previous myocardial infarction, episode of angina pectoris or stroke at baseline

### Relationships between adiposity indices and CV mortality

The cohort was observed for CV mortality from 1 August 1995 until 31 December 2015, mean observational time 17.7 ± 4.2 (mean ± SD) years, corresponding to 1080, 473.6 person-years. In this period, 13,195 (21.6%) participants died (6136 women, 7059 men), and of these, 4908 (37.2%) died from CV causes (2214 women, 2694 men). Annualised CV mortality rates were 3.9/1000 person-years for women and 5.3/1000 person-years for men, with higher absolute risk of CV death with increasing levels of all adiposity indices (Table 2a and 2b).

**Table 2a:** Unadjusted and age-adjusted associations between quartiles of traditional and non-traditional adiposity indices and CV death for women

	Women				
	*N* with available data	*N* of CV deaths	Incidence rates per 1000 patient years	Unadjusted HR (95% CI)	Age-adjusted HR (95% CI)
Waist (cm)	31,936	2214			
≤73.0	7464	192	1.36	1	1
73.1–80.0	8085	355	2.39	1.77 (1.49–2.11)	0.91 (0.76–1.08)
80.1–88.0	8549	681	4.47	3.35 (2.85–3.93)	0.97 (0.82–1.14)
≥88.1	7838	986	7.41	5.64 (4.83–6.59)	1.24 (1.06–1.45)
Waist-to- hip ratio	31,934	2214			
≤0.75	8022	214	1.43	1	1
0.76–0.79	7960	410	2.78	1.97(1.67–2.32)	1.12 (0.95–1.32)
0.80–0.83	7948	588	4.12	2.92 (2.50–3.42)	1.11 (0.94–1.30)
≥0.84	8004	1002	7.46	5.37 (4.63–6.23)	1.49 (1.28–1.73)
BMI (m/kg^2^)	31936	2214			
≤23.0	8078	302	2.02	1	1
23.1–25.5	7951	398	2.74	1.36 (1.17–1.58)	0.87 (0.75–1.01)
25.6–28.7	7950	614	4.30	2.15 (1.87–2.46)	0.87 (0.76–1.00)
≥28.8	7957	900	6.54	3.30 (2.90–3.76)	1.06 (0.93–1.20)
ICO	31,936	2214			
≤0.44	7996	140	0.92	1	1
0.45–0.49	7962	312	2.11	2.34 (1.91–2.85)	0.97 (0.79–1.19)
0.50–0.54	7983	630	4.44	4.95 (4.12–5.95)	1.02 (0.85–1.23)
≥0.55	7995	1132	8.49	9.68 (8.12–11.55)	1.28 (1.07–1.53)
eTBF (%)	31,936	2214			
≤21.8	7984	116	0.76	1	1
21.9–27.0	7995	310	2.09	2.84 (2.30–3.53)	1.15 (0.93–1.43)
27.1–32.2	7978	559	3.89	5.31 (4.34–6.49)	1.13 (0.93–1.39)
≥32.3	7979	1229	9.46	13.38 (11.05–16.22)	1.54 (1.26–1.87)
ABSI	31,936	2214			
≤0.69	7984	207	1.36	1	1
0.70–0.72	7984	324	2.20	1.62 (1.36–1.93)	0.92 (0.77–1.09)
0.73–0.75	7985	559	3.92	2.95 (2.52–3.46)	1.05 (0.89–1.23)
≥0.76	7983	1124	8.55	6.60 (5.69–7.66)	1.14 (1.14–1.15)

*CV* cardiovascular, *HR* hazard ratio, *CI* confidence interval, *WHR* waist-to-hip ratio, *BMI* body mass index, *ICO* index of central obesity, *eTBF* estimated total body fat, *ABSI* a body shape index

**Table 2b:** Unadjusted and age-adjusted associations between quartiles of traditional and non-traditional adiposity indices and CV death for men

	Men				
	*N* with available	*N* of CV deaths	Incidence rates per 1000 patient years	Unadjusted HR (95% CI)	Age-adjusted HR (95% CI)
Waist (cm)	29,080	2694			
≤86.0	7206	351	2.67	1	1
86.1–91.0	6891	497	4.04	1.52 (1.32–1.74)	1.06 (0.92–1.21)
91.1–97.0	7907	752	5.49	2.07 (1.82–2.35)	1.09 (0.96–1.24)
≥97.1	7076	1094	9.58	3.63 (3.22–4.10)	1.40 (1.24–1.58)
Waist-to-hip ratio	29,079	2694			
≤0.86	7324	286	2.14	1	1
0.87–0.89	7214	448	3.39	1.67 (1.44–-1.94)	1.04 (0.89–1.20)
0.90–0.93	7237	728	5.81	2.82 (2.46–3.24)	1.20 (1.05–1.38)
≥0.94	7304	1232	10.85	5.30 (4.65–6.03)	1.47 (1.29–1.68)
BMI (m/kg^2^)	29,080	2694			
≤24.1	7273	522	4.09	1	1
24.2–26.1	7298	650	5.07	1.24 (1.11–1.39)	0.99 (0.88–1.11)
26.2–28.4	7274	656	5.15	1.26 (1.12–1.41)	0.93 (0.83–1.05)
≥28.5	7235	866	7.07	1.73 (1.56–1.93)	1.20 (1.07–1.33)
ICO	29,080	2694			
≤0.48	7291	216	1.60	1	1
0.49–0.51	7241	466	3.56	2.26 (1.92–2.65)	1.20 (1.02–1.42)
0.52–0.55	7261	715	5.70	3.62 (3.11–4.22)	1.20 (1.03–1.39)
≥0.65	7287	1297	11.45	7.31 (6.33–8.45)	1.54 (1.33–1.79)
eTBF (%)	29,080	2694			
≤16.0	7281	200	1.47	1	1
16.1–19.4	7257	392	2.97	2.04 (1.72–2.41)	1.20 (1.02–1.42)
19.5–23.0	7272	655	5.17	3.58 (3.06–4.20)	1.20 (1.03–1.39)
≥23.1	7270	1447	13.26	9.32 (8.03–10.81)	1.54 (1.33–1.79)
ABSI	29,080	2 694			
≤0.75	7269	235	1.71	1	1
0.76–0.78	7271	403	3.04	1.77 (1.50–2.08)	0.96 (0.82–1.13)
0.79–0.80	7270	671	5.33	3.14 (2.71–3.65)	1.10 (0.94–1.28)
≥0.81	7270	1385	12.71	7.61 (6.62–8.74)	1.38 (1.19–1.59)

*CV* cardiovascular, *HR* hazard ratio, *CI* confidence interval, *WHR* waist-to-hip ratio, *BMI* body mass index, *ICO* index of central obesity, *eTBF* estimated total body fat, *ABSI* a body shape index

**Fig. 1:**
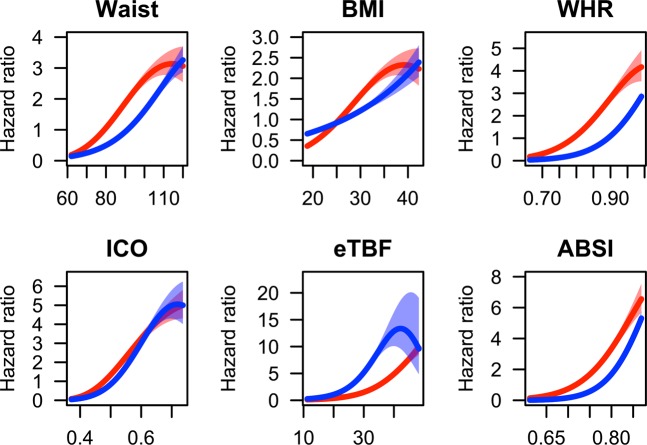
**a**–**f** The risk of cardiovascular death (hazard ratio with 95% confidence intervals) according to each unit of the adiposity indices, for men and women separately. **a** Waist circumference, **b** body mass index (BMI), **c** waist-to-hip ratio (WHR), **d** index of central obesity (ICO), **e** estimated total body fat (eTBF), and **f** a body shape index (ABSI) (blue: men; red: women)

All indices of adiposity, that is, general fat mass, abdominal fat mass and fat distribution were associated with CV mortality as illustrated in Fig. 1a–f, displaying HR for CV death by increasing levels of the individual adiposity index. The figures demonstrate a lower risk of CV death in the lowest ranges of the fat indices, and that the apparently most favourable levels of fat mass indices varied across sexes for most of the indices. For some indices, in particular eTBF in women, the risk of CV death seemed to level off at values corresponding to severe obesity. Of note is also the gender effect on CV mortality risk, in particular for eTBF, but also for WC and WHR. The CV mortality risk increases at lower levels of eTBF in men as compared to women, whereas for WC and WHR, the risk increases at slightly lower levels in women as compared to men.

The HR for CV death increased by quartiles of adiposity indices (Table 2a and 2b) in men as well as in women. In both genders, the non-traditional index eTBF showed the strongest association (e.g. 4th vs. 1st quartile HR in women 13.38 (95% CI: 11.05–16.22) and in men HR 9.32 (8.03–10.81). Of note is also the weaker association between the HR for CV death and traditional indices, in particular BMI in men. In an age-adjusted model (Table 2a and 2b), the magnitude of association was less for all indices, yet eTBF remained a strong index in men, whereas in women, WHR and eTBF appeared to be of equal strength. Sensitivity analyses excluding participants with high risk for CV death at baseline (known CV disease, diabetes and current smokers) did not alter the associations between the adiposity indices and CV death (data not shown).

Across gender and age strata (Table 3a and 3b), all adiposity indices were related to CV death, but the HRs were somewhat higher in the younger age group, also after age adjustment. Regardless of age stratum, BMI showed the weakest association with CV death, whereas eTBF and ABSI appeared to show the strongest association. After age adjustment however, in the elderly, all indices except BMI seemed to be associated with CV death by similar magnitude.

**Table 3a:** Unadjusted and age-adjusted associations between quartiles of the adiposity indices and CV death stratified by age in women

	Women (*n*=31,936)			
Age	19–59 years (*n* = 23,120)	19–59 years (*n* = 23,120)	60–79 years (*n* = 8816)	60–79 years (*n* = 8816)
Waist circumference (cm)	Unadjusted HR (95% CI)	Age-adjusted HR (95% CI)	Unadjusted HR (95% CI)	Age-adjusted HR (95% CI)
1st quartile	1	1	1	1
2nd quartile	1.77 (1.14–2.76)	1.24 (0.80–1.94)	0.91 (0.76–1.11)	0.86 (0.71–1.04)
3rd quartile	1.70 (1.07–2.69)	0.99 (0.62–1.57)	1.13 (0.95–1.35)	0.95 (0.80–1.13)
4th quartile	3.58 (2.35–5.46)	1.75 (1.14–2.69)	1.46 (1.24–1.73)	1.18 (1.00–1.40)
WHR				
1st quartile	1	1	1	1
2nd quartile	1.62 (1.01–2.60)	1.36 (0.85–2.19)	1.33 (1.11–1.58)	1.07 (0.90–1.28)
3rd quartile	2.05 (1.29–3.26)	1.42 (0.89–2.25)	1.37 (1.16–2.22)	1.05 (0.89–1.25)
4th quartile	4.05 (2.64–6.22)	2.36 (1.53–3.62)	1.89 (1.61–2.22)	1.40 (1.19–1.64)
BMI (kg/m^2^)				
1st quartile	1	1	1	1
2nd quartile	1.59 (1.04–2.46)	1.15 (0.74–1.77)	0.82 (0.70–0.96)	0.84 (0.71–0.98)
3rd quartile	1.66 (1.07–2.58)	0.96 (0.62–1.50)	0.88 (0.76–1.02)	0.86 (0.74–0.99)
4th quartile	2.89 (1.92–4.35)	1.49 (0.98–2.25)	1.07 (0.93–1.22)	1.02 (0.89–1.18)
ICO				
1st quartile	1	1	1	1
2nd quartile	1.94 (1.23–3.08)	1.28 (0.80–2.02)	1.00 (0.80–1.25)	0.91 (0.73–1.13)
3rd quartile	2.57 (1.63–4.05)	1.38 (0.87–2.19)	1.23 (1.01–1.51)	0.95 (0.78–1.13)
4th quartile	4.46 (2.88–6.89)	1.99 (1.28–3.10)	1.67 (1.37–2.03)	1.18 (0.97–1.43)
eTBF (%)				
1st quartile	1	1	1	1
2nd quartile	2.00 (1.23–3.23)	1.38 (0.85–2.24)	1.27 (1.00–1.61)	1.07 (0.84–1.36)
3rd quartile	2.90 (1.82–4.63)	1.63 (1.02–2.62)	1.41 (1.13–1.77)	1.02 (0.81–1.28)
4th quartile	5.88 (3.75–9.21)	2.67 (1.69–4.20)	2.21 (1.78–2.75)	1.37 (1.10–1.70)
ABSI				
1st quartile	1	1	1	1
2nd quartile	1.31 (0.82–2.11)	1.06 (0.66–1.70)	1.01 (0.84–1.22)	0.88 (0.73–1.06)
3rd quartile	2.39 (1.55–3.69)	1.62 (1.05–2.50)	1.27 (1.07–1.51)	0.97 (0.82–1.15)
4th quartile	3.72 (2.44–5.66)	2.16 (1.41–3.30)	2.00 (1.71–2.35)	1.33 (1.13–1.57)

*CV* cardiovascular, *HR* hazard ratio, *CI* confidence interval, *WHR* waist-to-hip ratio, *BMI* body mass index, *ICO* index of central obesity, *eTBF* estimated total body fat, *ABSI* a body shape index

**Table 3b:** Unadjusted and age-adjusted associations between quartiles of the adiposity indices and CV death stratified by age in men

	Men (*n*=29,080)			
Age	19–59 years (*n* = 21,237)	19–59 years (*n* = 21,237)	60–79 years (*n* = 7843)	60–79 years (*n* = 7843)
Waist circumference (cm)	Unadjusted HR (95% CI)	Age-adjusted HR (95% CI)	Unadjusted HR (95% CI)	Age-adjusted HR (95% CI)
1st quartile	1	1	1	1
2nd quartile	1.72 (1.25–2.37)	1.20 (0.87–1.64)	1.02 (0.88–1.19)	1.03 (0.88–1.20)
3rd quartile	2.01 (1.48–2.74)	1.20 (0.88–1.63)	1.15 (1.00–1.32)	1.06 (0.92–1.22)
4th quartile	4.12 (3.09–5.50)	2.10 (1.57–2.81)	1.41 (1.23–1.61)	1.29 (1.13–1.48)
WHR				
1st quartile	1	1	1	1
2nd quartile	1.62 (1.17–2.25)	1.10 (0.79–1.53)	1.06 (0.90–1.25)	1.01 (0.86–1.20)
3rd quartile	2.76 (2.04–3.75)	1.54 (1.13–2.09)	1.22 (1.04–1.42)	1.13 (0.96–1.32)
4th quartile	4.58 (3.40–6.15)	1.97 (1.46–2.67)	1.59 (1.38–1.84)	1.37 (1.18–1.59)
BMI (kg/m^2^)				
1st quartile	1	1	1	1
2nd quartile	1.42 (1.04–1.92)	1.09 (0.80–1.47)	0.97 (0.86–1.10)	0.97 (0.86–1.10)
3rd quartile	1.59 (1.18–2.13)	1.07 (0.80–1.44)	0.87 (0.77–0.98)	0.91 (0.80–1.03)
4th quartile	2.65 (2.02–3.49)	1.67 (1.27–2.20)	1.06 (0.94–1.19)	1.12 (0.99–1.26)
ICO				
1st quartile	1	1	1	1
2nd quartile	2.58 (1.85–3.62)	1.64 (1.17–2.30)	1.04 (0.86–1.25)	1.08 (0.89–1.29)
3rd quartile	2.83 (2.01–3.97)	1.44 (1.02–2.03)	1.20 (1.01–1.42)	1.09 (0.92–1.29)
4th quartile	6.65 (4.84–9.12)	2.86 (2.08–3.95)	1.57 (1.34–1.85)	1.31 (1.11–1.54)
eTBF (%)				
1st quartile	1	1	1	1
2nd quartile	2.34 (1.67–3.26)	1.53 (1.09–2.14)	1.04 (0.85–1.27)	1.08 (0.89–1.32)
3rd quartile	3.32 (2.40–4.58)	1.71 (1.23–2.37)	1.20 (1.00–1.44)	1.10 (0.92–1.32)
4th quartile	6.45 (4.70–8.86)	2.58 (1.87–3.56)	1.83 (1.54–2.17)	1.42 (1.20–1.68)
ABSI				
1st quartile	1	1	1	1
2nd quartile	1.25 (0.94–1.66)	0.94 (0.71–1.26)	1.07 (0.88–1.31)	0.95 (0.78–1.16)
3rd quartile	1.78 (1.35–2.34)	1.10 (0.83–1.45)	1.34 (1.12–1.61)	1.08 (0.90–1.30)
4th quartile	3.15 (2.41–4.12)	1.62 (1.23–2.12)	2.00 (1.68–2.37)	1.32 (1.11–1.58)

*CV* cardiovascular, *HR* hazard ratio, *CI* confidence interval, *WHR* waist-to-hip ratio, *BMI* body mass index, *ICO* gindex of central obesity, *eTBF* estimated total body fat, *ABSI* a body shape index

**Fig. 2:**
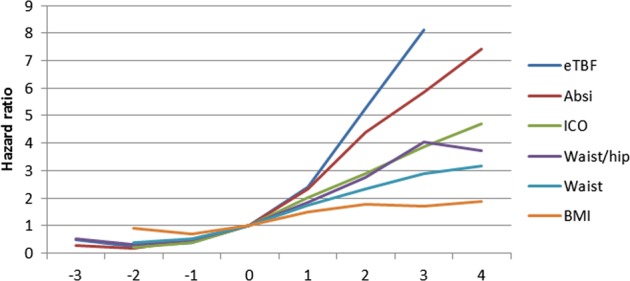
The risk of cardiovascular death (hazard ratio) according to standardised adiposity indices (standardised by sex-specific *z*-scores of each adiposity index). WHR: waist-to-hip ratio, BMI: body mass index, ICO: index of central obesity, eTBF: estimated total body fat, ABSI: a body shape index

The discriminative ability of the different indices as expressed by *C*-statistics is shown in Table 4. In both women and men, the discriminative ability of eTBF was robust (>0.7) and appeared to best capture the risk for CV death together with ICO in women and ABSI in men. When analysing sex-specific *z*-scores by individual adiposity indices, both eTBF and ABSI were strongly associated with CV death (Fig. 2).

**Table 4:** *C*-statistics for unadjusted models of the traditional and non-traditional adiposity indices in prediction of CV death

	*C*-statistics (95% confidence interval)	
	Women	Men
eTBF	0.725 (0.713–0.737)	0.711 (0.701–0.721)
ICO	0.704 (0.692–0.716)	0.682 (0.672–0.692)
ABSI	0.690 (0.678–0.702)	0.700 (0.690–0.710)
Waist circumference	0.663 (0.651–0.675)	0.626 (0.616–0.636)
WHR	0.659 (0.647–0.671)	0.664 (0.654–0.674)
BMI	0.622 (0.610–0.634)	0.551 (0.541–0.562)

*WHR* waist-to-hip ratio, *BMI* body mass index, *ICO* index of central obesity, *eTBF* estimated total body fat, *ABSI* a body shape index

In this large, population-based study with observation of individuals for more 17 years comprising more than one million person-years, we found that the non-traditional adiposity index eTBF was the strongest discriminator of CV death in both men and women. This index discriminated better than the traditional indices (BMI, WC and WHR), and on par with ICO in women and ABSI in men. To our knowledge, this is the first study to assess the discriminative property of the sex-specific adiposity index eTBF, a new and easy-to-calculate index that may be useful for clinicians for CV risk estimation.

The relationship between risk for CV mortality and overweight is complex. However, studies that have evaluated the association between obesity and life expectancy have shown that extreme obesity may shorten life expectancy up to 14 years [17] and that the obesity-associated mortality has increased over the past decades [18–20]. BMI is the most commonly used tool for clinicians to classify obesity. However, acknowledging the already mentioned limitations of BMI, it is debated whether BMI is an appropriate measure to identify obesity related CV risk [21, 22]. A more accurately defined risk score may help the health care provider to emphasise the importance of reduction of weight and body fat [23], and better indices of adiposity than BMI are thus needed. Assessment of fat mass by the gold-standard approach requires imaging techniques, which are impossible to implement in clinical practice due to high cost, radiation exposure, and long scan time. Surrogate indices of total adiposity like eTBF, which is highly correlated with total body fat content as measured by dual x-ray absorptiometry (DXA) [23, 24], is therefore an applicable tool for estimation of CV mortality risk associated with adiposity.

Previous analyses of non-traditional adiposity indices, including a former analysis of the present cohort with fewer years of follow-up [5], did not evaluate ABSI or eTBF, but in general, these studies support the notion that non-traditional indices of obesity are more strongly associated with CV mortality than BMI [8, 25, 26].

We found that measures of visceral fat such as WC and WHR, traditionally regarded to be better predictors of mortality than BMI, were weaker discriminators for CV mortality risk than eTBF. One reason could be that eTBF, unlike the others, is gender specific, and thus incorporates the gender disparity in adipose function [27–30] and distribution [31], that is, women tend to have higher levels of subcutaneous fat for a given BMI or WC [32]. This could also explain the higher mortality risk at lower eTBF levels in men seen in Fig. 1. Further, as the eTBF formula encompasses both WC and weight, it may reflect other important fat depots, for example, epicardial or intramuscular, which are missed when WC, the hip circumference or height alone or in combination are assessed. In accordance with this, a previous study from Iceland demonstrated that the amount of intermuscular fat in the thigh was associated with increased mortality in subjects aged 66–96 years [33]. The finding is in accordance with the present report showing that eTBF was a better discriminator for CV death than ICO in men and likely women (Table 4). However, ICO is a better predictor than BMI [5, 7, 8, 25, 26, 34] and ICO correlates well with the amount of visceral fat tissue by MRI [24]. Another potential explanation for our finding is the possibility that eTBF reflects different cardiometabolic risk than that expressed by the amount of visceral or total body fat, for example, risk related to the relative amount of the two fat compartments or gender. More extensive validation of eTBF in another population is necessary to verify our findings, and to investigate whether reduction of eTBF would translate into a lower CV risk.

The other two non-traditional adiposity indices, ABSI and ICO, were both more strongly associated with CV death than the established measures (BMI, WC and WHR). ABSI was derived from a US general population sample and incorporates WC and BMI, and thus requires the measurement of height, unlike eTBF [10]. High ABSI indicates that WC is higher than expected for a given height and weight and suggests abdominal obesity. We found that ABSI seemed to discriminate better than ICO in men, whereas in women, ICO was better than ABSI, although the confidence intervals were overlapping for both sexes (Table 4). These findings are in line with other studies of ABSI in both elderly [6] and middle-aged populations [35]. A gender disparity of ABSI is also in accordance with previous findings [36]. In men, ABSI was positively correlated to the fat mass index, and negatively correlated to DXA-measured fat-free mass index, whereas in women, ABSI was positively correlated to both fat mass index and fat-free mass index [36]. This discrepancy may reflect the difference in body composition between genders, with a tendency of accumulation of fat centrally in men, and in the gluteal–femoral region in women [37], and may explain why ABSI may not be able to identify or discriminate the hazardous abdominal visceral fat from other tissue as well in women as in men.

Age modified the association of all adiposity indices’ to CV mortality risk as indicated in Table 2a and 2b and in the age-strata analysis (Table 3a and 3b), underscoring that age is a risk modifier, in line with other studies [6, 35, 38]. Nevertheless, also in the elderly, we find a “dose response” between increasing quartiles of all adiposity indices and risk of CV death, except for BMI. This may be explained by BMI’s lacking ability to capture body composition and body fat distribution: as age advances, lean body mass decreases and fat mass increases with a preferential distribution in the abdominal region [39].

As shown in Figs. 1 and 2, the relationship between all the adiposity indices and risk of CV death were close to linear up to higher levels of adiposity, in women as well as in men. Interestingly, at higher levels of all adiposity indices, except for BMI in men, the associated risk of CV death seemed to level off. This may potentially be explained by competing risks, “the obesity paradox” [40], or be an artefact due to lower sample size in the higher ranges of adiposity.

The strengths of this study are the large number of participants, the population-based prospective design, the objectively measured body height, weight, and waist and hip circumference and the completeness of the data set, enhancing the generalisability of our results. Furthermore, the homogeneity of the population is of importance, since ethnicity and racial differences in body composition influence the associations of anthropometric measures with CV outcomes. However, our findings may not be generalised beyond Caucasians, since our dataset does not include African Americans or Asians. The principal limitation of our study, as of other registry-based studies, is the restricted possibility to address all potential confounding factors.

In this population-based study with more than one million person-years of observation, the gender-specific adiposity index eTBF was associated with CV death in men as well as in women, and more so than other non-traditional and traditional adiposity indices. eTBF reflects the amount of total body fat, is easily calculated and may thus be a suitable clinical tool for assessment of obesity-associated CV risk.

## Supplementary information

Supplementary Table S1
