# Supplementary material for: Comparison of the associations between non-traditional and traditional indices of adiposity and cardiovascular mortality: an observational study of one million person-years of follow-up
Source: Int J Obes (Lond). 2019 Mar 29;43(5):1082–92. doi: 10.1038/s41366-019-0353-9 (PMC6760583; doi:10.1038/s41366-019-0353-9)
Supplement: Supplementary file 1 — Supplementary Table S1 [file 41366_2019_353_MOESM1_ESM.docx]

Supplementary Table S1a and S1b: Correlations (Spearman Rho) between the adiposity indices for women (a) and men (b) separately.

a

|  | Waist | WHR | BMI | ICO | eTBF | ABSI |
| --- | --- | --- | --- | --- | --- | --- |
| Waist | 1 | 0.780, <0.001 | 0.852, <0.001 | 0.960,  <0.001 | 0.889,  0.001 | 0.625,  0.001 |
| WHR | 0.780, <0.001 | 1 | 0.519,  <0.001 | 0.782, <0.001 | 0.853, <0.001 | 0.764, <0.001 |
| BMI | 0.852, <0.001 | 0.519,  <0.001 | 1 | 0.857, <0.001 | 0.616, <0.001 | 0.185, <0.001 |
| ICO | 0.960,  <0.001 | 0.782, <0.001 | 0.857, <0.001 | 1 | 0.924, <0.001 | 0.619, <0.001 |
| eTBF | 0.889,  0.001 | 0.853, <0.001 | 0.616, <0.001 | 0.924, <0.001 | 1 | 0.858, <0.001 |
| ABSI | 0.625,  0.001 | 0.764, <0.001 | 0.185, <0.001 | 0.619, <0.001 | 0.858, <0.001 | 1 |

Abbreviations: CV: cardiovascular, HR: hazard ratio, CI: confidence interval, WHR: waist-hip ratio, BMI: body mass index, ICO: index of central obesity, eTBF: estimated total body fat, ABSI: a body shape index.

b

|  | Waist | WHR | BMI | ICO | eTBF | ABSI |
| --- | --- | --- | --- | --- | --- | --- |
| Waist | 1 | 0.802, <0.001 | 0.800, <0.001 | 0.919, <0.001 | 0.884, <0.001 | 0.482, <0.001 |
| WHR | 0.802, <0.001 | 1 | 0.524, <0.001 | 0.815, <0.001 | 0.872, <0.001 | 0.653, <0.001 |
| BMI | 0.800, <0.001 | 0.524, <0.001 | 1 | 0.809, <0.001 | 0.564, <0.001 | -0.046, <0.001 |
| ICO | 0.919, <0.001 | 0.815, <0.001 | 0.809, <0.001 | 1 | 0.922, <0.001 | 0.472, <0.001 |
| eTBF | 0.884, <0.001 | 0.872, <0.001 | 0.564, <0.001 | 0.922, <0.001 | 1 | 0.754, <0.001 |
| ABSI | 0.482, <0.001 | 0.653, <0.001 | -0.046, <0.001 | 0.472, <0.001 | 0.754, <0.001 | 1 |

Abbreviations: CV: cardiovascular, HR: hazard ratio, CI: confidence interval, WHR: waist-hip ratio, BMI: body mass index, ICO: index of central obesity, eTBF: estimated total body fat, ABSI: a body shape index.
